# The Etiology of Total Knee Arthroplasty Failure Influences on Improvement in Knee Function: A Follow-Up Study

**DOI:** 10.3390/jcm13247672

**Published:** 2024-12-16

**Authors:** Sandy Weis, Lisa Seifert, Moritz Oltmanns, Farouk Khury, Ralf Bieger, Martin Faschingbauer

**Affiliations:** 1 Department of Orthopedic Surgery, University of Augsburg, 86159 Augsburg, Germany; 2 Clinic for Anesthesiology, University of Augsburg, 86159 Augsburg, Germany; 3 Department of Orthopedic Surgery, University of Ulm, 89081 Ulm, Germany; moritz.oltmanns@rku.de; 4 Rambam Health Care Campus, Orthopedic Surgery, Haifa 3109601, Israel; 5 Schön Klinik München Harlaching, 81547 Munich, Germany; ralfbieger@hotmail.com; 6 Department of Orthopedics, Klinik Penzing, Wiener Gesundheitsverbund, 1140 Vienna, Austria

**Keywords:** tka, tka revision, outcome revision tka

## Abstract

**Background:** Outcomes following total knee arthroplasty (TKA) revisions are variable, and it is hypothesized that the underlying cause of primary TKA failure impacts postoperative outcomes. This study analyzes the results of TKA revisions seven years after surgery, in relation to the etiology of primary failure and other influencing factors. A previous study conducted in 2013 examined the same cohort of patients three months after revision surgery. **Methods:** From the original study, 97 patients were followed up, and 49 patients were eligible for inclusion. Patients were classified into four groups: “periprosthetic infection” (PPI), “aseptic loosening,” “instability,” and “arthrofibrosis.” Outcomes were analyzed using established scores (the Knee Society Score (KSS), New Knee Society Score (nKSS), and the Western Ontario and McMaster Universities (WOMAC) index) and considering factors such as age, gender, BMI, and ASA classification. The outcomes were analyzed using three time intervals: “Outcome Short” (preoperative to 3 months postoperative); “Outcome Long” (preoperative to 7 years postoperative); and “Delta PostOP” (3 months postoperative to 7 years postoperative). **Results:** Significant improvements were observed in all time intervals, especially in the “Outcome Short” (*p* < 0.001) and “Delta PostOP” (*p* < 0.001) periods. The “instability” and “aseptic loosening” groups showed better outcomes in terms of range of motion and knee scores than the “arthrofibrosis” and “infection” groups (*p* < 0.05). A lower BMI and an ASA status of II were associated with better outcomes (*p* < 0.05). Women also showed superior results in the nKSS (*p* < 0.05). **Discussion:** Patients with “aseptic loosening” and “instability” had the best long-term outcomes. Lower BMI and better ASA status also correlated with improved results. These findings can inform patients of the potential outcomes of revision surgery, thereby facilitating informed decision-making.

## 1. Patients and Methods

### 1.1. Study Design and Participants

This study is based on the study by Bieger et al. [1] from 2013, which examined the potential influencing factors on the clinical results following revision knee replacement surgery. The study design is observational and retrospective.

#### 1.1.1. Inclusion and Exclusion Criteria

**Figure 1:**
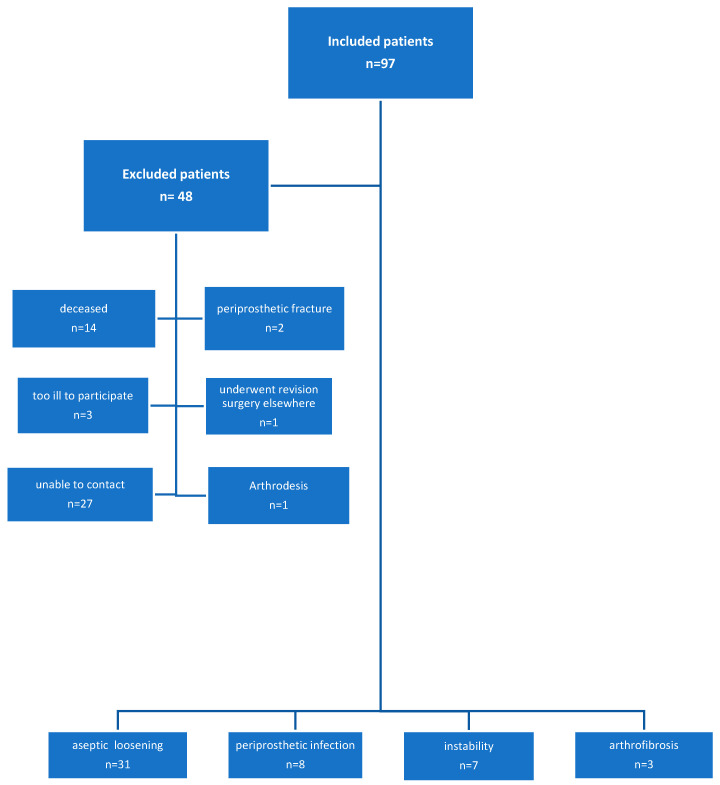
Included and excluded patients.

A total of 97 consecutive patients who underwent knee revision surgery between 2006 and 2009 at our institution were included in the study [2,3]. The mean follow-up period was 29.2 ± 9.9 months (range 12–51 months) [4]. All patients underwent revision total knee arthroplasty (TKA) using a modular semi-constrained prosthesis (Legion, Smith & Nephew, Dover, MA, USA). The Knee Society Score (KSS) was recorded preoperatively, at three months, and annually postoperatively. In 22 cases, the revision surgery was necessitated by a confirmed periprosthetic infection. In 75 patients, a one-stage TKA exchange was performed due to aseptic failure [5]. Aseptic loosening was observed in 55 patients, instability in 13 cases, and arthrofibrosis in 7 cases (Figure 1). Incomplete changes, such as conversion from unicompartmental knee arthroplasty (UKA) to bicondylar prostheses [6] and isolated component changes, as well as arthrodesis, were excluded from the study.

#### 1.1.2. Patient Demographics

Thus, 97 eligible patients were included in the study, with a mean age of 68.0 ± 9.2 years (range 44–87 years) and a mean body mass index (BMI) of 30 ± 6.2 kg/m^2^ (range 17.3–41.9 kg/m^2^). The mean interval between the initial surgery and the TKA revision was 68.1 months. In addition to the preoperative diagnosis, the patient’s age, body mass index (BMI), gender, and American Society of Anesthesiologists (ASA) classification were recorded.

### 1.2. Follow-Up and Outcome Measures

In the current study, all patients were contacted by mail. Fourteen patients were deceased, two patients had a periprosthetic fracture, three patients were too ill to participate, one patient underwent another revision surgery in another institution, and another patient had an arthrodesis. Twenty-seven patients were unable to be contacted. Consequently, a total of 49 patients were included in the study. The patients exhibited an average age of 73.5 years (range 52–92 years) and a mean BMI of 30.1 kg/m^2^ (range 21–58 kg/m^2^). The mean interval between primary surgery and revision TKA was 58.3 months (range 6–234). Of the 49 patients included in the study, 63.2% (31/49) were female, while 36.8% (18/49) were male.

#### Outcome Measures

The follow-ups included three questionnaires as a clinical examination.

- Knee Society Score (KSS): the KSS was recorded preoperatively, at three months postoperatively, and annually thereafter.
- New Knee Society Score (nKSS): This includes an Objective Knee Score (OKS) and an activity score. The Objective Knee Score (OKS) is a composite score, which includes the visual analogue scale for pain, and the functional score, which incorporates the walking distance and patient’s ability to climb stairs. The activity section of the nKSS includes a comprehensive range of everyday and leisure activities, allowing for the recording of the diverse lifestyles of younger patients.
- Western Ontario and McMaster Universities Score (WOMAC): used for assessing pain, stiffness, and physical function in patients undergoing joint replacement.

Three time intervals were defined:

- Outcome Short (OS): preoperative—3 months postoperative (PostOP1)
- Outcome Long (OL): preoperative—7 years postoperative (PostOP2)
- Delta PostOP (∆P): 3 months postoperative—7 years postoperative

The clinical examination yielded the following results: range of motion (ROM) and mediolateral and anteroposterior stability (grade 0: 0–5 mm; grade 1: 5–10 mm; grade 2: 10–15 mm; and grade 3: >15 mm). X-rays were taken (short knee radiographs anteroposterior and sagittal, sunrise view), and the full leg length in a standing position was measured (hip–knee–ankle angle). The evaluation of the X-rays, the clinical examination, and the evaluation of the questionnaires were carried out by the same experienced surgeon to ensure consistency in the clinical assessment.

### 1.3. Categorization of Revision Etiology

As in our initial study from 2013, patients were classified into four categories based on the etiology of primary prosthesis failure [7]: “aseptic loosening” (31 patients); “periprosthetic infection” (8 patients) [8]; “instability” (7 patients); and “arthrofibrosis” (3 patients). The four etiologies [9] were evaluated for significant changes in the following characteristics: range of motion (ROM); flexion/extension; mediolateral and anteroposterior stability; maximum walking distance; and the three questionnaires (KSS, nKSS, and WOMAC). Prior to surgery, the groups exhibited differences in their indications. As a result, aseptic loosening and instability were distinguished primarily by their mediolateral and anteroposterior instability, arthrofibrosis, and infection, which were evident in a significant deficit in range of motion, flexion, and extension.

In addition to the primary etiology of failure, the following factors were examined for their influence on outcomes [10,11,12,13]:

- Age;
- Gender;
- Body Mass Index (BMI);
- American Society of Anesthesiologists (ASA) classification.

### 1.4. Statistical Analysis

The *t*-test was employed for the statistical evaluation of the study, with the objective of comparing the pre- and postoperative KSS values within the subgroups. The KSS values were compared between the subgroups using one-factor analysis of variance and the *t*-test. Due to the limited number of patients diagnosed with arthrofibrosis, these results were excluded from the analysis of variance. The same tests were employed to ascertain the alterations in the KSS values from the preoperative to the postoperative periods. A linear regression analysis was conducted to identify factors associated with pre- and postoperative KSS values and the overall extent of improvement. The level of significance was set at *p* < 0.05. The statistical analysis was conducted using the program PASW Statistics (PASW, version 18, IBM, Armonk, NY, USA).

The data were collected using Microsoft Excel for Mac 2011, version 14.5.2 (Microsoft Corporation, Redmond, WA, USA). The statistical evaluation was created using the IBM SPSS Statistics program, version 24 (IBM Corporation, Armonk, NY, USA). The mean, standard deviation, and range were determined to represent the central tendencies of metric characteristics. The *t*-test for paired samples was employed to demonstrate the existence of significant differences in the data collected at disparate time points, namely preoperatively, three months postoperatively, and seven years postoperatively.

The Mann–Whitney U test was utilized to contrast the central tendencies between the four groups. Additionally, the Wilcoxon test was applied to identify significant differences within the groups at different points in time. In order to identify further influencing factors, the Mann–Whitney U test and simple linear regression analyses were employed. A significant difference was assumed at *p* < 0.05.

## 2. Results

### 2.1. Entire Patient Collective

A summary of all four groups demonstrated a statistically significant improvement in all characteristics in the short-term outcome. This improvement was also observed in the long-term outcome, except for the function score which demonstrated no significant improvement. All except the range of motion (ROM) exhibited a statistically significant deterioration in ∆P.

### 2.2. Comparison of the Results of the Four Indication Groups

Each group was analyzed individually in each time period (Table 1).

#### 2.2.1. Aseptic Loosening

The group of patients who underwent revision surgery due to aseptic loosening demonstrated a significant postoperative improvement in the short-term outcome, as measured by the total Knee Society Score (KSS), knee score, and function score. However, a significant deterioration was also observed in ∆P.

#### 2.2.2. Instability

The instability group exhibited comparable alterations to those observed in the loosening group.

#### 2.2.3. Arthrofibrosis

This group of patients demonstrated no significant improvement or deterioration over the entire observation period. However, there was a discernible trend towards enhanced outcomes in the short- and long-term, accompanied by a tendency towards deterioration in ∆P.

#### 2.2.4. Infection

The infection group patients demonstrated superior outcomes at both the short-term and long-term follow-up periods. However, between the two follow-up appointments, there was a decline in the observed results.

### 2.3. Comparison of the Four Indication Groups

The different groups were compared with each other in each time period (Table 2, Table 3 and Table 4).

#### 2.3.1. Arthrofibrosis

In comparison to the group of aseptic loosening, the preoperative range of motion (ROM) (*p* = 0.01) and the knee score 7 years postoperative (*p* = 0.04) are found to be significantly lower. A non-significant trend is observed when the medians of the two groups are compared, indicating that patients with aseptic loosening achieve better values than patients with arthrofibrosis. The group of patients with instability demonstrated a significant improvement in range of motion before surgery (*p* = 0.03) and in the knee score seven years after surgery (*p* = 0.03) compared to patients with arthrofibrosis. There were no significant differences between the arthrofibrosis and infection groups.

#### 2.3.2. Instability

Compared to the group of infection, there are significant differences in the preoperative and seven years postoperative results in favor of the instability group: the preoperative characteristics ROM (*p* = 0.01), function score (*p* = 0.03) and the ROM 7 years postoperative (*p* = 0.01). Both three months postoperatively and seven years postoperatively, a non-significant trend can be seen towards the instability group (vs. the infection group). No significant differences can be found between the aseptic loosening and instability groups.

#### 2.3.3. Aseptic Loosening

The two groups differed significantly in preoperative characteristics, including range of motion (*p* < 0.0001), total Knee Society Score (*p* < 0.0001), and function score (*p* < 0.0001), in comparison to the group of infection. Seven years postoperatively, the aseptic loosening group exhibited significantly higher ROM (*p* = 0.01) and knee score (*p* = 0.04) results than the infection group. With the exception of the New Knee and New Function Score, there is a non-significant trend in favor of the aseptic loosening group for the remaining parameters.

### 2.4. Comparison of Other Influencing Factors

#### 2.4.1. Age

The present study employed a linear regression analysis to investigate the influence of patient age at the time of revision surgery on surgical outcome. The analysis revealed no statistically significant association between these variables (*p* < 0.05).

#### 2.4.2. Gender

The present study indicates a non-significant trend whereby women tend to achieve superior postoperative outcomes compared to men. However, the results of the nKSS (*p* = 0.04) demonstrate that women do, in fact, achieve better outcomes than men.

#### 2.4.3. BMI

The results of this study demonstrate that patients with a lower BMI exhibited significantly superior outcomes, respectively, in total KSS (*p* < 0.0001), knee score (*p* = 0.01), and WOMAC score (*p* = 0.03) at three and seven years postoperatively, in comparison to patients with a higher BMI.

#### 2.4.4. ASA

Patients classified as ASA II demonstrated significantly superior outcomes seven years postoperatively for the total KSS (*p* = 0.02) compared to patients classified as ASA III or IV. For the remaining parameters, a non-significant trend (*p* > 0.05) for patients classified as ASA II can be observed.

## 3. Discussion

### 3.1. Discussion of the Results of the Entire Patient Collective

The present study, which recorded medium-term clinical results after rTKA with a minimum follow-up of seven years, demonstrated that the parameters collected in both the first and second examination intervals (Short and Long Outcome) exhibited significant improvement. However, in the interval between three months post-op and seven years later, the results exhibited deterioration in certain areas [14]. When considered individually, each indication group exhibited similar results to the entire patient collective [15]. The results of the study indicate that there was an improvement in the first two study periods (preoperatively to three months/seven years postoperatively) and a subsequent worsening in the post-op interval (three months postoperatively to seven years postoperatively). A meta-analysis by Shen et al. examined the outcome after rTKR in 33 scientific articles involving a total of 1356 patients [16]. Significant improvements were observed in the Knee Society Score (KSS), function score, and range of motion from preoperative to postoperative results. Additionally, newer studies demonstrated higher postoperative values than older ones. The authors propose that technological improvements in prosthesis design, improvements in surgical techniques, better patient selection, and improved postoperative patient management may all be contributing factors.

In their study, which had an average follow-up period of six years, Gil-Martínez et al. [17,18] demonstrated that the operation led to improved mobility in the knee joint, better KSS results, better function, and higher patient satisfaction. The mean preoperative knee score was 43.75 (present study: 52.33), while the mean postoperative score was 76.58 (present study: 61.18). There was a statistically significant improvement between the preoperative scores and the scores at the end of follow-up. The mean preoperative function score was 52.68 (present study: 46.43) and was 77.92 postoperatively (present study: 55.31), representing a statistically significant improvement. Both in the present study and in Gil-Martínez’s study, the number of cases was relatively low (Gil-Martínez: 41 patients, present study: 49 patients). In a similar vein, patients presenting with both aseptic and septic failure of the primary prosthesis were also included in the present study. However, it is important to note that in Gil-Martínez’s study, only aseptic failure (30 cases) and septic failure (11 cases) were included, which may explain the poorer outcomes observed in our study with regard to the knee score and function score.

Deehan et al. [19] also demonstrated that the KSS of 86 patients exhibited a significant improvement at three and twelve months postoperatively. Five years after the operation, the patients reported a reduction in pain levels compared to before the operation. However, the activity level of the patients was found to be reduced. The decline in activity levels observed in patients several years postoperatively, compared to three months postoperatively, is likely due to the increasing age of the patients and the associated restricted mobility or increase in comorbidities. Similar results to those reported by Deehan et al. were also found in our study. Seven years postoperatively, many patients achieved poorer results than three months postoperatively. It is possible that the observed decline in walking distance and activity levels, as well as the reduction in knee joint mobility and stability, can be attributed to several factors. These include the natural progression of age, the potential increase in comorbidities, and the inevitable wear and tear of the prosthesis.

In conclusion, it can be postulated that patients with an indication for rTKA may benefit significantly from this procedure. Improved mobility, reduced pain, and enhanced function of the knee joint can be expected from a total knee replacement. Nevertheless, over time, the results deteriorate once more. The improvements observed in the initial follow-up examinations (three months postoperatively in the present study) were reversed seven years after the operation.

### 3.2. Discussion of the Results Between the Indication Groups

The comparison between the four indication groups reveals not only individual significant differences but also a trend indicating that patients in the “arthrofibrosis” and “periprosthetic infection” groups have a poorer outcome after seven years than patients in the “aseptic loosening” and “instability” groups [20,21,22]. This is particularly evident in regard to the range of motion (ROM), flexion, and Knee Society Score (KSS). The results three months postoperatively demonstrate no significant differences between the groups. No significant difference or clear trend can be determined between the groups “arthrofibrosis” and “periprosthetic infection.” The same applies to the comparison of the groups “aseptic loosening” and “instability.” Patients with the indication of “aseptic loosening” had the best results three months and seven years after the operation. However, it can be demonstrated that all patients benefited from the revision surgery and showed better results postoperatively than preoperatively [23].

In conclusion, patients who undergo revision surgery due to aseptic loosening of the prosthesis achieve the best postoperative outcomes. Patients with stiffness as a reason for revision surgery achieve poorer outcomes. In patients with an infection of the prosthesis, several authors have reached different conclusions. Poorer (see present study), similar, and better results, compared to patients with aseptic failure of TKA, can be demonstrated. Nevertheless, the authors concur that a uniform approach to managing infection in TKA is essential to enhance the likelihood of favorable outcomes.

### 3.3. Discussion of the Other Influencing Factors

The present study did not identify any influence of the age of the patients at the time of the revision operation on the outcome [24,25]. Additionally, there was a non-significant trend indicating that women tend to achieve better postoperative results than men. However, only the results of the New Knee Society Score demonstrated that women achieve better results than men. A lower BMI is associated with significantly better total KSS, knee score, and function score results three months postoperatively, as well as better WOMAC score results seven years postoperatively, compared to patients with a higher BMI. Regarding the ASA classification, patients who were preoperatively assigned to group 1 and thus classified as ASA II demonstrated significantly better results than patients in group 2 with an ASA classification III or IV.

### 3.4. Limitations

The present study is limited by the relatively small number of patients included (49/97), due to a high rate of loss to follow-up. Elderly, multimorbid patients with rTKA are at an increased risk of loss to follow-up. Furthermore, some of the selected subgroups consist of fewer than ten patients, which precludes the generalizability of the collected data. A further limitation of the present study is that it was conducted as a unicentric study. Consequently, however, it includes a more homogeneous group of study participants. Another limitation of our study is the inter-individual perception of pain, different demands on the knee joint, and different lifestyles and basic attitudes of the patients, which affected the answers to the questionnaires [26]. One potential solution to this issue is for the patient and examiner to complete the questionnaires jointly. This could help to reduce ambiguity and lead to more objective and specific results. While other studies focus on the period immediately after the operation, or a maximum of two years postoperatively (5–30), this study has a long mean follow-up of 100.3 months (minimum 83 months, maximum 115 months).

## 4. Conclusions

With regard to the disparate outcomes associated with distinct etiologies, the findings of this study can facilitate more effective preoperative patient education. The current investigation revealed that rTKA represents a challenging procedure for patients and physicians alike. While the immediate postoperative result is highly encouraging, it is important to note that the outcomes diminish significantly over an extended follow-up period. Furthermore, our findings demonstrate that there are significant differences in the (short and long) follow-ups, contingent upon the underlying revision indications. These findings assist in the education of patients preoperatively regarding the realistic expectations of the outcome of rTKA.

## Figures and Tables

**Table 1:** Comparison of the results of the four indication groups. This table shows the results of the Wicoxon test for patients who underwent total knee arthroplasty replacement surgery due to loosening of the prosthesis. Significant *p* values are color coded. 1: preOP; 2: postOP1 (3 months postOP); 3: postOP2 (7 years postOP).

	Instability			Loosening			Arthrofibrosis			Infection		
	Median	Median	*p*-Value	Median	Median	*p*-Value	Median	Median	*p*-Value	Median	Median	*p*-Value
OS: preOP(1)–postOP1(2)	1	2		1	2		1	2		1	2	
ROM (degrees)	100	95	1	100	102.5	0.18	55	100	-	80	95	0.05
Total KSS (points)	102	165	0.02	113	169	<0.001	81	145	0.25	33	153.5	0.01
-Knee Score (points)	55	87	0.02	52	89	<0.001	62	85	0.25	38	83.5	0.01
-Function Score (points)	55	80	0.02	60	80	<0.001	50	60	0.50	10	70	0.01
OL: preOP(1)–postOP2(3)	1	3		1	3		1	3		1	3	
ROM (degrees)	100	110	0.63	100	110	0.03	55	100	0.50	80	87.5	0.09
Total KSS (points)	102	121	0.09	113	123	0.15	81	100	1	33	88.5	0.25
-Knee Score (points)	55	63	0.08	52	69	0.06	62	30	0.50	38	53.5	0.55
-Function Score (points)	60	60	0.28	60	55	0.78	50	70	0.75	10	40	0.06
∆P: postOP1(2)–postOP2(3)	2	3		2	3		2	3		2	3	
ROM (degrees)	95	110	0.25	102.5	110	0.96	100	100	-	95	87.5	0.13
Total KSS (points)	165	121	0.02	169	123	<0.001	145	100	0.25	153.5	88.5	0.02
Knee Score (points)	87	63	0.02	89	69	<0.001	85	30	0.25	83.5	53.5	0.02
Function Score (points)	80	60	0.03	80	55	<0.001	60	70	0.50	70	40	0.06

**Table 2:** Comparison between the group of aseptic loosening and the respective three other groups. This table shows the results of the Mann–Whitney U test for patients with revision total knee arthroplasty surgery in the aseptic loosening and the other three groups. Significant values are colored. A larger median means better results; the only exception is the WOMAC score, where a larger median means a worse result.

	Loosening (Median)	Vs. Arthrofibrosis (Median)	*p*-Value	Vs. Infection (Median)	*p*-Value	Vs. Instability (Median)	*p*-Value
Preoperative							
ROM (degrees)	100	55	0.01	80	<0.001	100	0.29
Total KSS (points)	113	81	0.26	33	0.01	102	0.21
-Knee Score (points)	52	62	0.78	38	0.16	55	0.41
-Function Score (points)	60	50	0.33	10	<0.001	55	0.08
Outcome short							
ROM (degrees)	102.5	100	0.61	95	0.10	95	0.07
Total KSS (points)	169	145	0.21	153.5	0.12	165	0.36
-Knee Score (points)	89	85	0.12	83.5	0.17	87	0.51
-Function Score (points)	80	60	0.23	70.0	0.18	80	0.48
Outcome long							
ROM (degrees)	110	100	0.12	87.5	0.01	11	0.41
Total KSS (points)	123	100	0.17	88.5	0.13	121	0.97
-Knee Score (points)	69	30	0.04	53.5	0.04	63	0.84
-Function Score (points)	55	70	0.93	40	0.27	60	0.89
Total New KSS (points)	124	109	0.29	90	0.22	112	0.42
-New Knee Score(points)	40	40	0.88	42	0.99	50	0.84
-New Function Score (points)	58	53	0.41	59	0.38	66	0.56
WOMAC (points)	71	110	0.15	74	0.68	113	0.44

**Table 3:** Comparison between the group of instability and the groups of arthrofibrosis and infection. This table shows the results of the Mann–Whitney U test for patients with total knee replacement surgery in the instability and the other two groups. Significant values are colored. A larger median means better results; the only exception is the WOMAC score, where a larger median means a worse result.

	Instability (Median)	Vs. Arthrofibrosis (Median)	*p*-Value	Vs. Infection (Median)	*p*-Value
Preoperative					
ROM (degrees)	100	55	0.03	80	0.01
Total KSS (points)	102	81	0.83	33	0.19
-Knee Score (points)	55	62	0.83	38	0.61
-Function Score (points)	55	50	1	10	0.03
Outcome short					
ROM (degrees)	95	100	0.50	95	0.67
Total KSS (points)	165	145	0.38	153.5	0.28
-Knee Score (points)	87	85	0.18	83.5	0.34
-Function Score (points)	80	60	0.38	70	0.34
Outcome long					
ROM (degrees)	100	100	0.12	87.5	0.01
Total KSS (points)	121	100	0.27	88.5	0.34
-Knee Score (points)	63	30	0.03	53.5	0.12
-Function Score (points)	60	70	1	40	0.40
Total New KSS (points)	112	109	0.73	90	0.42
-New Knee Score (points)	50	40	0.73	42	0.91
-New Function Score (points)	66	53	0.73	59	0.73
WOMAC (points)	113	110	0.57	74	0.86

**Table 4:** Comparison between the group of arthrofibrosis and the group of infection. This table shows the results of the Mann Whitney U test for patients with total knee replacement surgery in the arthrofibrosis and the infection group. Significant values are colored. A larger median means better results at that point in time. The only exception is the WOMAC score, where a larger median means a worse result.

	Arthrofibrosis (Median)	Vs. Infection (Median)	*p*-Value
Preoperative			
ROM (degrees)	55	80	0.92
Total KSS (points)	81	33	0.28
-Knee Score (points)	62	38	0.92
-Function Score (points)	50	10	0.19
Outcome short			
ROM (degrees)	100	95	0.75
Total KSS (points)	145	153.5	0.78
-Knee Score (points)	85	83.5	0.78
-Function Score (points)	60	70	0.50
Outcome long			
ROM (degrees)	100	87.5	0.92
Total KSS (points)	100	88.5	0.63
-Knee Score (points)	30	53.5	0.28
-Function Score (points)	70	40	0.92
Total New KSS (points)	109	90	1
-New Knee Score (points)	40	42	0.84
-New Function Score (points)	53	59	0.92
WOMAC (points)	110	74	0.22

## Data Availability

The data is available to the authors and can be viewed on request.
